# Seeded Vapor Phase Growth of Fe-Intercalated Fe_3_GeTe_2_ Nanoplates with Large Coercive Field

**DOI:** 10.1021/acs.nanolett.6c01803

**Published:** 2026-08-06

**Authors:** Yueai Lin, Zixiao Shi, Fan Fei, Yangchen He, Ying Wang, Daniel A. Rhodes, Jun Xiao, David A. Muller, Song Jin

**Affiliations:** † Department of Chemistry, 5228 University of Wisconsin−Madison, Madison, Wisconsin 53706, United States; ‡ Department of Chemistry and Chemical Biology, 5922 Cornell University, Ithaca, New York 14853, United States; § Department of Materials Science and Engineering, University of Wisconsin−Madison, Madison, Wisconsin 53706, United States; ∥ Department of Electrical and Computer Engineering, University of Wisconsin−Madison, Madison, Wisconsin 53706, United States; ⊥ Department of Physics, University of Wisconsin−Madison, Madison, Wisconsin 53706, United States; # School of Applied & Engineering Physics, Cornell University, Ithaca, New York 14853, United States; 7 Kavli Institute at Cornell for Nanoscale Science, Cornell University, Ithaca, New York 14853, United States

**Keywords:** 2D magnetic materials, Fe_3_GeTe_2_, electron ptychography, Fe intercalation, RMCD

## Abstract

Two-dimensional (2D) magnets provide a versatile platform for exploring emergent quantum phases and developing next-generation spintronic devices. Despite this potential, high-throughput chemical vapor deposition (CVD) of ternary phase 2D magnets remains a significant challenge and is rarely explored. Here, we report a seeded-CVD method to synthesize nanoplates of a 2D magnetic material, Fe_3_GeTe_2_ (FGT), with lateral sizes of 10 μm and thicknesses of 20–80 nm. Synthesized nanoplates exhibit high Curie temperature (*T*_c_ ∼ 206 K) and large coercive field (∼1 T) based on reflective magnetic circular dichroism (RMCD) measurements. Cross-sectional scanning transmission electron microscopy and multislice electron ptychography directly reveal widespread and 3D-inhomogeneous Fe intercalation within the vdW gaps that is quantified to be Fe_3+*x*_GeTe_2_ with *x* ≈ 0.4, which resolves the atomic structural origins of the magnetic enhancement. These results enable a scalable route to synthesize 2D ternary magnet Fe_3_GeTe_2_ directly for property studies and device integration.

## Graphical abstract

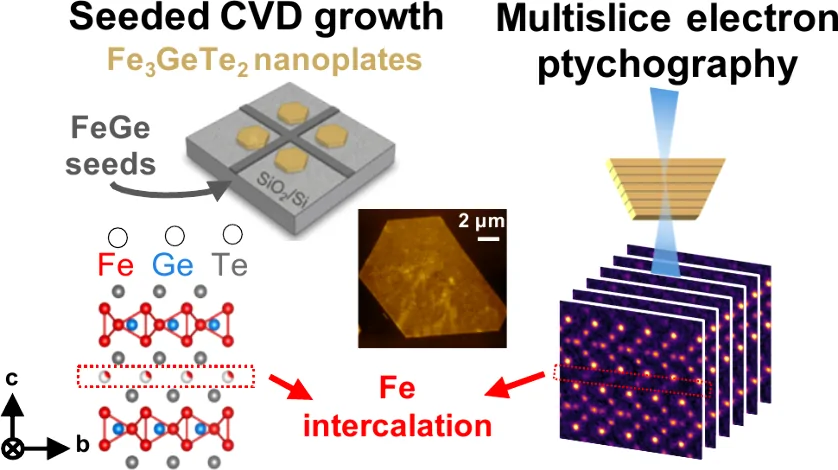

Two-dimensional (2D) or van der Waals (vdW) magnets provide ideal platforms for exploring emergent physics and spintronic devices with an atomic-scale control. Unlike their bulk counterparts, which often lose magnetic order at atomic scales due to thermal fluctuations, 2D magnets can maintain their properties down to the monolayer limit and possess flexibility for property tuning.^1^ The discovery of layer-dependent magnetism in vdW crystals such as CrI_3_ and Cr_2_Ge_2_Te_6_ has established a robust framework for tuning long-range ferromagnetism through dimensionality and stacking symmetry.^2,3^ This frontier has since expanded to encompass exotic magnetic phases in systems like the multiferroic NiI_2_ and the intrinsic topological insulator MnBi_2_Te_4_, enabling the exploration of noncollinear spin textures and topological quantum phenomena at the atomic limit.^4,5^ Among 2D ferromagnets, Fe_3_GeTe_2_ (FGT) stands out as a good candidate from the family of Fe_*n*_GeTe_2_ for spintronic devices due to its metallic nature, relatively high Curie temperature (*T*_c_ = 160–210 K),^6–8^ and its ability to host topological skyrmions.^9–12^ Fe_3_GeTe_2_ stacks in AB′ sequence, different from the ABC-stacked (3R) Fe_4_GeTe_2_ and Fe_5_GeTe_2_.^13–15^ Increasing the Fe ratio of the Fe_*n*_GeTe_2_ family strengthens the interatomic exchange interactions, thereby stabilizing a more robust ferromagnetic ground state and consequently elevating *T*_c_. For bulk single crystals of Fe_*n*_GeTe_2_, *T*_c_ ranges from 150 K for Fe_2.64_GeTe_2_ grown via self-flux, 270 K for Fe_4_GeTe_2_ grown via stoichiometric solid-state reaction, to 310 K for Fe_5_GeTe_2_ grown via chemical vapor transport (CVT).^13,14,16^ Thin flakes of Fe_*n*_GeTe_2_ exhibit slightly lower *T*_c_, but they offer versatile tuning parameters for manipulating magnetism at the nanoscale, such as using intercalation, doping, ionic gating, and strain engineering to increase the Curie temperature. In contrast, hole doping through increasing Fe vacancy^17^ and electron doping through Co substitution^18,19^ have been reported to suppress ferromagnetism. Moreover, a recent study has demonstrated the first example of a spin glass state in 2D materials on Fe-intercalated FGT flakes owing to disordered spins of inhomogeneously distributed intercalated Fe atoms.^20^ Therefore, synthesizing Fe-intercalated Fe_3_GeTe_2_ would be valuable for expanding the understanding of spin glass behaviors in 2D magnets.

Ideal Fe_3_GeTe_2_ crystallizes in a centrosymmetric hexagonal layered structure, with the space group *P*6_3_/*mmc* (Figure 1a).^15^ Each quintuple layer consists of a covalently bonded Fe_3_Ge slab sandwiched by atomic Te sheets, with adjacent layers coupled via vdW interactions. Inside the Fe_3_Ge slab, Fe atoms occupy two distinct sites: the Fe atoms at the dumbbell positions are Fe^3+^, and those located in the Fe–Ge plane are Fe^2+^.^15^ The unit cell is composed of 2 layers, arranged in the 2H-like AB′ stacking sequence (Figure 1b). Previous literature reports that Fe intercalation can naturally occur in Fe_3_GeTe_2_ and an isostructural compound Fe_3_GaTe_2_, as well as in Fe_5_GeTe_2_.^21–23^ The interstitial Fe atoms partially occupy the octahedral interstitial sites inside the vdW gap (Figure 1c).

**Figure 1:**
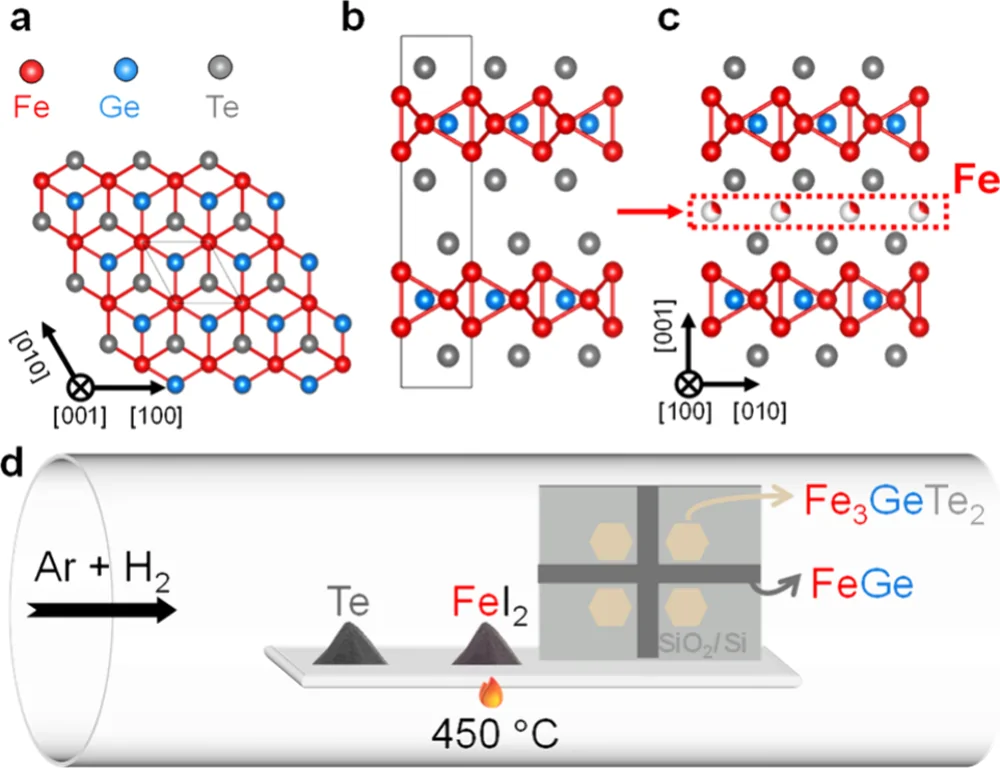
Crystal structure of Fe_3_GeTe_2_ (FGT) and the experimental setup of controllable chemical vapor deposition growth. **a**) Crystal structure of ideal FGT viewed along [001]. **b,c**) Side views along the [100] showing the layered structure of FGT **b**) without and **c**) with additional interstitial Fe atoms partially occupying the vdW gap (red box). **d**) Experimental setup of CVD growth of Fe_3_GeTe_2_ nanoplates using substrates seeded with FeGe spread out in a criss-cross pattern (illustrated in the inset).

To date, mechanical exfoliation of bulk single crystals of FGT has been the standard approach to preparing few-layer flakes of FGT, but this method is limited by the low yield, lack of scalability, and random thickness control, making it unsuitable for larger-scale applications. In contrast, vapor-phase growth provides a scalable alternative for producing high-quality thin films or nanostructures. Chemical vapor deposition (CVD), a standard vapor growth method, is widely used to synthesize mono- to few-layer thin films and heterostructures of 2D materials, particularly transition-metal dichalcogenides.^24^ However, CVD growth of ternary compounds is difficult and rarely reported, due to their complex crystal structures and the often more favorable growth of binary compounds in nonconfined space. The few exceptions include metal oxyhalide,^25–28^ metal phosphorus chalcogenides,^29^ and the Mn–Bi–Te family (MnBi_2_Te_4_ and MnBi_4_Te_7_),^30^ which were often synthesized via space-confined CVD methods. Here, we report a novel seeded-CVD method to synthesize Fe_3_GeTe_2_ nanoplates with lateral sizes of ten micrometers and thicknesses of tens of nanometers. We further employ electron ptychography and magnetic property measurements to correlate the structure with properties of these CVD-grown Fe_3_GeTe_2_ nanoplates, which were found to contain significantly inhomogeneously intercalated Fe atoms in their vdW gaps.

Our CVD setup for synthesizing Fe_3_GeTe_2_ plates on Si/SiO_2_ substrates is shown in Figure 1d. On the Si/SiO_2_ wafer, FeGe precursor powder^31^ was dropcast into a criss-cross pattern as the seeded Ge source. At the center of the furnace, a 6 cm-long alumina boat was placed with Te powder at the upstream end, FeI_2_ powder at the center, and the Si/SiO_2_ substrate at the downstream end. The furnace was preheated to target temperature under Ar and H_2_ flow, and the reaction took place at around 450 °C for 10 min before the furnace was opened and cooled down naturally (see details in Materials and Methods in SI).

As-grown Fe_3_GeTe_2_ nanoplates found near the FeGe seeded regions throughout the substrates were mostly hexagonal, with typical lateral sizes of 3–15 μm (Figure 2a,b). While most plates grew horizontally, some exhibited vertical growth (Figure 2b); this vertical orientation specifically facilitates sample transfer. The typical thickness of hexagonal Fe_3_GeTe_2_ nanoplates is 15–80 nm, occasionally more than 150 nm. A representative Fe_3_GeTe_2_ nanoplate is 10 μm wide and 30 nm thick (Figure 2c). The plates show good compositional uniformity, confirmed by the energy-dispersive X-ray spectroscopy (EDS) ratios obtained from multiple distinct samples distributed across the substrate. The CVD-synthesized product on substrates was characterized by powder X-ray diffraction (PXRD) (Figure 2d), which revealed multiple intense peaks that match the (00*l*) peaks of Fe_3_GeTe_2_, confirming the product phase and preferential growth of flat Fe_3_GeTe_2_ nanoplates.^7^ Before acquiring the PXRD pattern, we intentionally removed unreacted FeGe precursor particles by drop-casting polycarbonate (PC) solution onto the growth substrates then peeling off the dried PC film. This step effectively removed the precursor particles without destroying the nanoplate products, because nanoplates have stronger bonds with the wafer.

**Figure 2:**
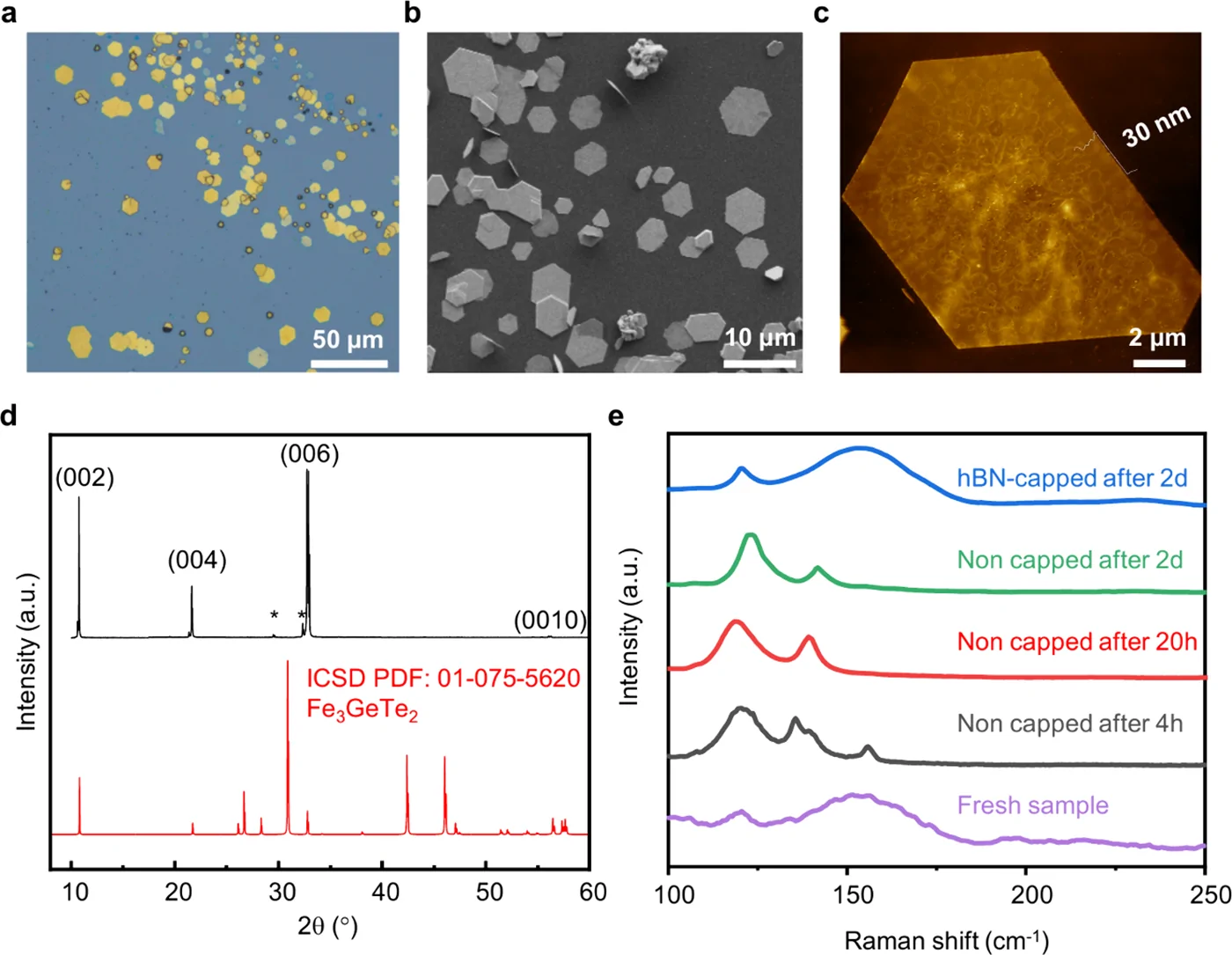
Structural characterizations of Fe_3_GeTe_2_ nanoplates. **a**) Optical microscope image and **b**) SEM image of as-grown Fe_3_GeTe_2_ nanoplates. **c**) AFM image of a representative 30 nm thick Fe_3_GeTe_2_ nanoplate. **d**) The powder X-ray diffraction pattern of Fe_3_GeTe_2_ on the growth substrate showing the (00*l*) peaks in comparison with the standard PXRD pattern (ICSD PDF# 01-075-5620). “*” marks minor impurity peaks from Ge and FeTe_2_. **e**) Raman spectra comparing hBN-protected and unprotected FGT nanoplates after various intervals of ambient exposure.

Small amounts of impurities with different shapes were observed away from the seeded regions due to the favorable growth of binary compounds, as hexagonal FGT nanoplates formed preferably around the seeded region. Due to the low density of these byproduct phases, they often fall below the detection limit of PXRD. We used scanning electron microscope (SEM)/EDS and Raman spectroscopy to identify these impurity phases that include tetragonal FeTe nanoplates, hexagonal FeTe_2_ nanoplates, and orthorhombic FeTe_2_ nanowhiskers (SI Figure S1). The shape difference makes it easy to distinguish FGT nanoplates from these byproducts. The only other hexagonal byproduct phase, FeTe_2_ nanoplates, typically formed thick (>100 nm) and large plates (>20 μm) far from the seeded region. A very minor PXRD peak in Figure 2d corresponds to the FeTe_2_ impurity phase. The composition of the byproduct phases can be tuned by adjusting the hydrogen flow: under a high hydrogen flow (20 sccm), rectangular nanoplates of tetragonal FeTe became the sole byproducts. This preferential growth of FeTe is attributed to the enhanced reduction of FeI_2_ and increased flux of Fe-containing vapor.

We optimized the synthesis by evaluating different ways of drop-casting the FeGe precursor powder (Figure S2a). When the FeGe/IPA suspension was spread on the whole Si/SiO_2_ substrate, FGT nanoplates with an average lateral size of ∼5 μm formed preferentially near large precursor powder clusters (Figure S2b). However, this distribution gave limited insights on whether it is local solid conversion or a vapor-phase transport reaction. Therefore, the FeGe precursor was confined to a single circular seeded area at the center of the wafer. Small FGT nanoplates grew in the seeded zone, while large FGT nanoplates formed outside the seeded boundary (Figure S2c,d). This spatial separation confirms that the growth is mediated by a gas-phase reaction, where gaseous species diffuse outward from the FeGe precursor to nucleate larger FGT plates. These findings also suggest that the diffusion distance of Ge-containing species determines the region of FGT deposition. Furthermore, the observation that the bare substrate promotes the growth of larger FGT nanoplates suggests that maximizing the perimeter of the seeded region is essential to promote the growth of larger nanoplates. Therefore, we spread dry FeGe powder into a ‘criss cross’ pattern to maximize the boundary area available for diffusion-limited growth while providing a controlled, localized precursor flux, thereby increasing the yield of FGT nanoplates (Figure S2a).

We further confirm the phase and stability of Fe_3_GeTe_2_ nanoplates with Raman spectroscopy (Figure 2e). It is worth mentioning that previous literature reports conflicting Raman spectra of Fe_3_GeTe_2_, with peaks at ∼120 cm^–1^ and 155 cm^–1^ or two peaks at ∼120 cm^–1^ and 144 cm^–1^.^32–35^ We hypothesize that this discrepancy of Raman spectra originates from environmental degradation or laser-induced damage, which has been systematically investigated in prior studies using Raman spectroscopy and X-ray photoelectron spectroscopy (XPS).^36–38^ To test this hypothesis, we compared the Raman spectra of freshly synthesized nanoplates (<1 h ambient exposure), samples aged in air for up to 2 days, and as-grown nanoplates encapsulated with exfoliated hexagonal boron nitride (hBN) nanosheets. The fresh FGT nanoplates and the hBN-capped nanoplates both show two peaks at ∼120 cm^–1^ and 155 cm^–1^. The intensity is inherently low due to the metallic nature of FGT. After 4 h of ambient exposure, a new peak at 144 cm^–1^ emerged. In 2 days, the peak at 155 cm^–1^ vanished, and the intensities of 120 cm^–1^ and 144 cm^–1^ peaks increased 5-fold in intensity due to oxidation. This behavior matches previous reports on oxidized FGT, for which FGT oxidizes to form Fe_2_O_3_ and GeO_2_, releasing Te from its original bonding to generate the 144 cm^–1^ peak.^36,38^ The similar Raman spectra obtained confirm again that the 155 cm^–1^ peak is the signature of pristine FGT, the 144 cm^–1^ peak is a marker for oxidation, and hBN encapsulation provides robust protection of FGT nanoplates.

We used scanning transmission electron microscopy (STEM) high angle annular dark field (HAADF) imaging along the top-down view to confirm the crystal structure of these Fe_3_GeTe_2_ nanoplates. The FGT nanoplates were transferred from the SiO_2_/Si substrate to Au TEM grids through a conventional polycarbonate (PC) stamp method.^39^ An atomic resolution HAADF-STEM image shows typical hexagonal structure and matches well with Fe_3_GeTe_2_ along its [001], [110], and [210] axes (Figure 3a, Figure S3). The corresponding fast Fourier transform (FFT) pattern (Figure 3b) shows a single set of spots, confirming the single crystallinity of the CVD-grown flake with a calculated lattice parameter of 4.11 Å, consistent with the value reported for FGT crystals grown via CVT.^40^ Interestingly, some weak superlattice spots appear at the midpoints between the major spots, with periodicity twice the unit cell length. The exact origin of the superlattice spots is unclear, but we have determined that they are not due to an ordered superlattice (discussed later). To further confirm these high-resolution STEM images are representative of the entire Fe_3_GeTe_2_ nanoplate, large field-of-view STEM energy dispersion spectrum (EDS) mapping shows the Fe, Ge, and Te elements are uniformly distributed within the whole nanoplate (Figure 3c). We also examined the edge surface and middle regions of FGT nanoplates with large field-of-view atomic resolution HAADF imaging (Figure S4), which indicates that only a single phase of Fe_3_GeTe_2_ is present (excluding some clearly oxidized regions).

**Figure 3:**
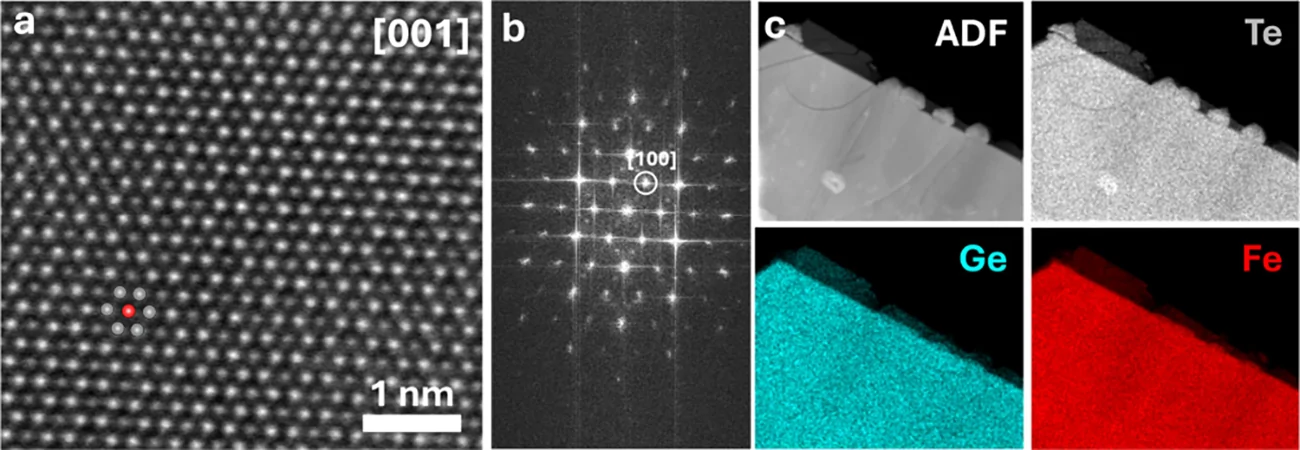
STEM imaging of the atomic structure of Fe_3_GeTe_2_ nanoplates. **a**) HAADF STEM image along [001], showing the periodic arrangement of Fe_3_GeTe_2_ nanoplates. **b**) FFT of the HAADF image along [001]. **c**) Large field-of-view STEM EDS mapping of Fe_3_GeTe_2_ nanoplates.

After confirming the phase, we characterized the magnetic properties of individual Fe_3_GeTe_2_ nanoplates by employing reflective magnetic circular dichroism (RMCD) and found unusually large coercive fields compared to exfoliated flakes measured in previous reports.^6,7^ First, the rectangular hysteresis loops at 10 and 100 K confirm the robust ferromagnetism of Fe_3_GeTe_2_ nanoplates, with strong perpendicular magnetic anisotropy (Figure 4a,b). In addition, we did not observe a clear thickness dependence of the coercive field among four FGT nanoplates with thicknesses of 36, 49, 38, and 15–20 nm (Figure 4d); this is likely because thickness effects are most pronounced in the monolayer-to-few-layer regime,^6,7,37^ and the thickness variation among these samples is relatively small. Notably, the measured coercive field of a 38 nm thick Fe_3_GeTe_2_ nanoplate (flake 3) reaches 9000 Oe at 10 K and ∼4500 Oe at 100 K. These coercive fields are significantly larger than the 3000 Oe reported at 105 K for a 48 nm thick FGT flake exfoliated from a bulk single crystal (Table S1).^6,7^ Temperature-dependent RMCD measurements of flake 1 show that the coercive field decreases and the hysteresis loops become less rectangular at higher temperatures (Figure 4c), indicating a transition from a hard magnet to a soft magnet. At 224 K, the hysteresis completely vanishes due to the transition to a paramagnetic state. The *T*_c_ is estimated to be 206 K through fitting of the temperature-dependent RMCD measurements (Figure S5), which is consistent with the behaviors observed for Fe-intercalated bulk single Fe_3_GeTe_2_ crystals grown via chemical vapor transport. It was reported that Fe intercalation can raise the *T*_c_ from 160 K in nonintercalated FGT to 210 or 230 K in partially intercalated Fe_3_GeTe_2_.^21^ The enhanced *T*_c_ and coercive fields observed in these CVD-synthesized FGT nanoplates suggest that Fe intercalation might be present.

**Figure 4:**
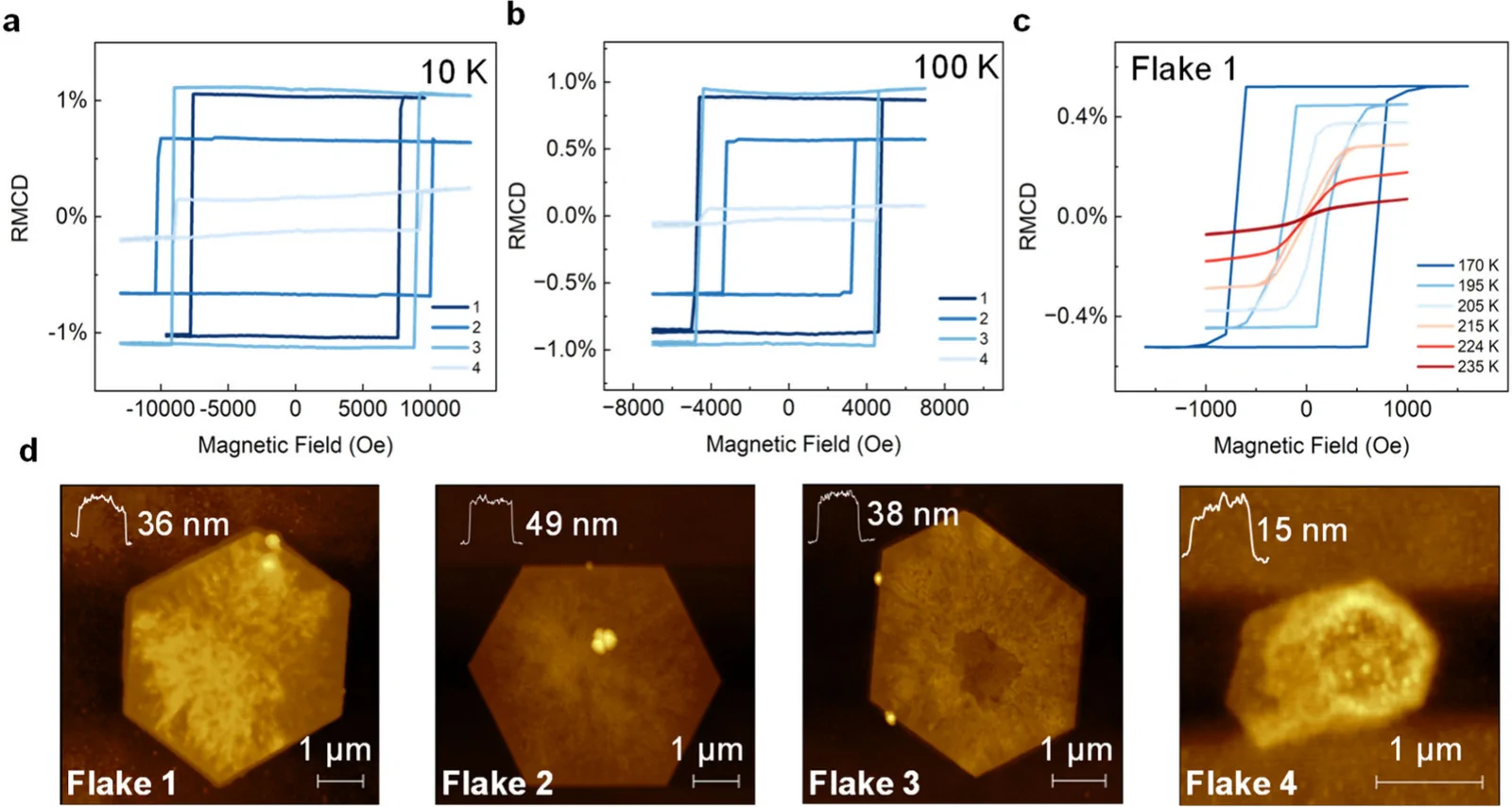
Magnetic properties of four representative Fe_3_GeTe_2_ (FGT) nanoplates. **a**,**b**) RMCD sweeps for FGT nanoplates at 10 K (**a**) and 100 K (**b**). **c**) Temperature-dependent RMCD measurements of flake 1. **d**) Atomic force microscopy (AFM) topography images of the corresponding FGT nanoplates with thickness line profiles. Labels 1–4 denote individual nanoplates evaluated during the measurement.

To confirm and measure the Fe intercalation in the vdW gap of Fe_3_GeTe_2_, we performed multislice electron ptychography (MEP).^41,42^ Beyond confirming the atomic structure with 2.5 times better lateral resolution than HAADF imaging, the key structural information MEP can provide to our system is the three-dimensional (3D) distribution of intercalated Fe atoms in the vdW gap.^43^ Since the depth resolution of HAADF is around 8–10 nm with a 30 mrad convergence angle, the HAADF image can be regarded as a 2D projection of the object. Comparatively, MEP provides a 2–3 nm depth resolution and can visualize the 3D inhomogeneity of the intercalated atom distribution.^43^ To directly visualize intercalated Fe atoms in 3D, we imaged the cross sections of Fe_3_GeTe_2_ nanoplates using MEP along two different crystallographic directions, [210] and [110] (Figure 5a). Because MEP reconstructs the sample as a series of depth slices, it allows us to follow individual atomic columns and possible vacancies along the electron beam direction. In these cross-sectional views, the regular Fe, Ge, and Te columns form the host Fe_3_GeTe_2_ lattice, while the extra Fe atoms occupy octahedral interstitial sites in the vdW gaps (Figure 5b,c).

**Figure 5:**
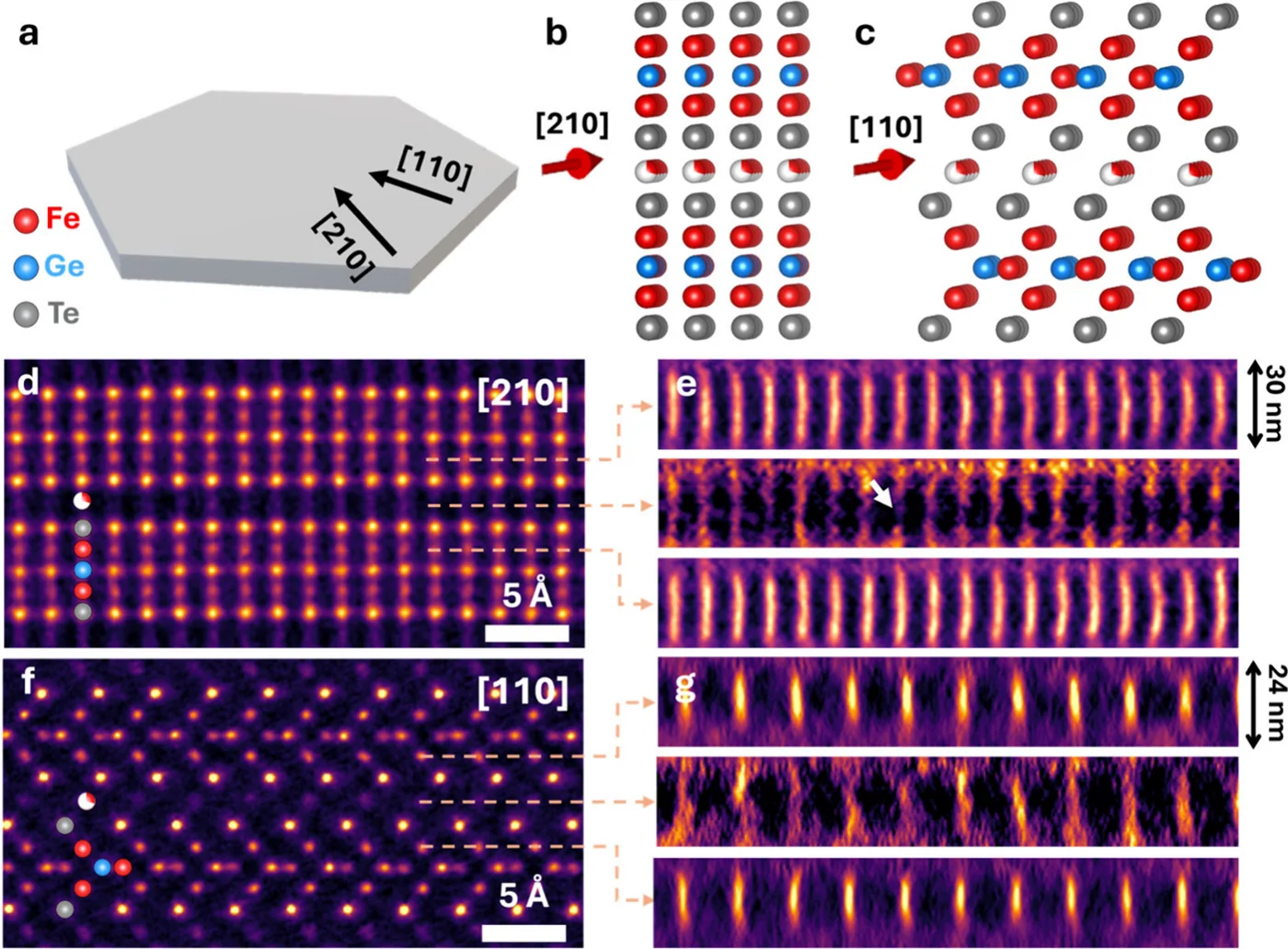
Ptychography reconstruction showing the inhomogeneous 3D distribution of intercalated Fe atoms in FGT nanoplates. **a)** Schematic illustration of the imaging orientations within the hexagonal FGT nanoplate, including the [210] and [110] cross-sectional viewing orientations. The atomic spheres are color-coded by element for consistency with the corresponding images in subsequent panels. **b, c)** Atomic models showing the Fe-intercalated FGT along the **b**) [210] and **c**) [110] directions. Red arrows mark the direction of the depth profiles into the planes from which depth profiles were extracted in **e** and **g**. **d–g)** MEP 1 nm slice images and depth profiles of Fe_3+*x*_GeTe_2_ nanoplates, showing the 3D inhomogeneous distribution of intercalated Fe by comparing the unit cell (framework) structural vs the interlayer Fe atom columns along the **(d, e)** [210] and **(f, g)** [110] directions. The structural Fe atom columns in the unit cell are continuous and have strong intensity; the Fe atom columns in the interlayers (highlighted with white arrows) are often discontinuous and have low intensity.

For the [210] orientation (Figure 5b,d,e), a 1 nm-thick MEP slice resolves the atomic columns in projection, while the corresponding depth profiles show how the intensity of selected columns changes through the sample thickness. The total reconstructed thickness is about 30 nm. Near both surfaces, we observe a 3–4 nm amorphous or oxidized layer, likely caused by FIB preparation and air exposure (Figure S6). Therefore, the quantitative analysis of Fe occupancy was performed only in the intact crystalline interior, excluding these surface regions.

The unit cell Fe columns in the Fe_3_GeTe_2_ host remain continuous and show nearly constant intensity along the beam direction (see top and bottom panels of Figure 5e), indicating that the host lattice is well preserved, without obvious Fe deficiency throughout this thickness. In contrast, the intercalated Fe columns in the van der Waals gap show much weaker and highly nonuniform intensities (middle panel of Figure 5e). Some of these intercalated Fe columns are discontinuous along the depth direction, with dark gaps corresponding to 2–3 nm regions where intercalated Fe atoms are absent, as highlighted in the white arrows of Figure 5e. A similar inhomogeneous distribution is observed in the [110] orientation (Figure 5f,g), where the intercalated Fe columns also show a weak intensity and obvious gaps along the depth direction. The much lower intensity of these intercalated columns compared to the unit cell Fe columns also shows that the population of intercalated Fe atoms (*x*) is relatively low.

We quantified the intercalated Fe concentration by comparing the summed intensity of the intercalated Fe columns with that of the unit cell Fe columns in the crystalline region, since the MEP signal is linear in the number of atoms/column.^41,42^ This quantitative histogram analysis (Figures S7, S8) gives an average excess Fe content of *x* ≈ 0.4, corresponding to a composition of approximately Fe_3+0.4_GeTe_2_. Detailed quantification procedures are described in the Supporting Information (Section: “Quantification of Interstitial Fe Content”). These results, along with Figures 5e,g, show that Fe intercalation in these CVD-grown Fe_3+*x*_GeTe_2_ nanoplates is three-dimensionally inhomogeneous rather than forming a long-range ordered superlattice. Consequently, the superlattice spots observed in Figure 3b do not originate from the same mechanism as those in Fe_1/4_TaS_2_.^44^

We hypothesize that Fe intercalation induces the relatively high *T*_c_ and large coercive field of CVD-grown FGT nanoplates, as demonstrated in previous studies.^17,21,45^ This enhancement has been attributed to electron doping, which elevates the magnetic anisotropy energy (MAE) by expanding the Fermi surface area of the electron pockets, a consequence of the electronic band shift.^17^ Fe intercalation also strengthens the itinerant ferromagnetism by reinforcing the intralayer ferromagnetic interaction.^45^ In addition, intercalated Fe atoms in the vdW gap can introduce additional interlayer exchange paths that enhance the Curie temperature and can locally couple antiferromagnetically with the ferromagnetic FGT layers, producing exchange bias-like AFM/FM pinning.^21^ This pinning increases the energy barrier for magnetization reversal and provides a plausible mechanism for an enhanced coercive field.

So far, Fe_3_GeTe_2_ has only been reported to exist in the centrosymmetric AB′ stacking, precluding ferroelectricity, which requires the breaking of inversion symmetry. The HAADF-STEM images further revealed some stacking anomalies in FGT nanoplates that include a noncentrosymmetric 3R phase and a more complex noncentrosymmetric macroscopic 3R-like stacking (Figures S9, S10). The unit cell of this new macroscopic 3R-like stacking is composed of nine layers of FGT, organized into three subblocks that are ABC stacked containing three AB′A-stacked layers in each subblock (Figure S9c). The whole plates exhibit three distinct stackings separated by two clear horizontal boundaries perpendicular to the *xy*-plane, and the stacking is laterally homogeneous (Figures S9a, S10). The top region is ABC-stacked, analogous to 3R-stacked transition metal dichalcogenides,^46^ the middle of the plate has the previously reported AB′ stacking, and the bottom of the plate exhibits a more complex macroscopic 3R-like stacking. The cross-sectional HAADF-STEM images (Figure S9b,c) show the stacking near boundaries, with the proposed FGT unit cell models overlaid. These diverse stackings suggest that FGT can host multiple phases. It is worth noting that this stacking anomaly is not a universal phenomenon; out of five distinct nanoplates examined via cross-sectional TEM, the anomaly was observed in only a single sample. Because both the 3R and 3R-like configurations break bulk inversion symmetry, these noncentrosymmetric phases are expected to be second-harmonic generation (SHG) active. Specifically, the observation of the noncentrosymmetric 3R phase suggests that intentionally synthesizing phase-pure 3R-FGT via molecular beam epitaxy may be a feasible route to realizing multiferroicity in FGT.

In conclusion, we developed a seeded CVD method to synthesize Fe-intercalated Fe_3+*x*_GeTe_2_ nanoplates. By employing cross-sectional STEM and electron ptychography, we confirm and quantify the Fe intercalation in the vdW gaps and reveal structural stacking anomalies in Fe_3_GeTe_2_ nanoplates. Magnetic characterization reveals a higher coercive field and Curie temperature in these Fe_3+*x*_GeTe_2_ nanoplates, as compared to previously reported bulk crystals of Fe_3_GeTe_2_, due to the Fe intercalation (*x* ≈ 0.4). These results underscore the efficacy of seeded CVD for the growth of ternary 2D materials. The crystalline nanoplates of Fe_3+*x*_GeTe_2_ provide a robust material platform for the study of physical properties and development of next-generation spintronic devices.

## Supplementary Material



## Notes

Details of synthesis, structural characterization, and reflective magnetic circular dichroism (RMCD) measurements of Fe_3_GeTe_2_ (FGT) nanoplates; additional optical microscope images and illustration of spatial distribution of reaction (by)­products; temperature dependence of the RMCD signal of the FGT flake with a model fit used to extract the Curie temperature; additional top-down STEM images and cross-sectional STEM images of the (210) plane and (110) plane and along [110] axis of FGT nanoplates and in larger field-of-view at different regions; cross sectional MEP images of FGT nanoplates along the [110] axis, together with the methods and results used to quantify intercalated Fe within FGT; structural layer stacking anomalies in FGT nanoplates; a table comparing the magnetic properties of various FGT samples (PDF)
