# Supplementary material for: Seeded Vapor Phase Growth of Fe-Intercalated Fe3GeTe2 Nanoplates with Large Coercive Field
Source: Nano Lett. 2026 Aug 6;26(32):10660–7. doi: 10.1021/acs.nanolett.6c01803 (PMC13495943; doi:10.1021/acs.nanolett.6c01803)
Supplement: Supplementary file 1 [file nl6c01803_si_001.pdf]

Supporting Information for

Seeded vapor phase growth of Fe-intercalated Fe_3GeTe_2 nanoplates with large coercive field

Yueai Lin[1], Zixiao Shi[2], Fan Fei[3], Yangchen He[4], Ying Wang[4,3,5], Daniel A. Rhodes[3,5], Jun Xiao[3,4,5], David A. Muller[6,7], Song Jin[1]*

[1]Department of Chemistry, University of Wisconsin–Madison, Madison, Wisconsin 53706, United States

[2]Department of Chemistry and Chemical Biology, Cornell University, Ithaca, New York 14853, United States

[3]Department of Materials Science and Engineering, University of Wisconsin–Madison, Madison, Wisconsin 53706, United States

[4]Department of Electrical and Computer Engineering, University of Wisconsin–Madison, Madison, Wisconsin 53706, United States

[5]Department of Physics, University of Wisconsin-Madison, Madison, Wisconsin 53706, USA

[6]School of Applied & Engineering Physics, Cornell University, Ithaca, New York 14853, United States

[7]Kavli Institute at Cornell for Nanoscale Science, Cornell University, Ithaca, New York 14853, United States

*****Corresponding Author. Email: jin@chem.wisc.edu

Materials and Methods

Synthesizing FeGe precursors

The FeGe precursor powder was synthesized following the literature.[1] Stochiometric amount (1:1) of iron (Sigma-Aldrich 99.9%, <10 μm particle size) and germanium powder (Sigma-Aldrich 99.999%, −100 mesh) were weighed out to a total mass of 0.25 g. The powder was ground in an agate mortar and pestle for ~10 min, then transferred to a 6 mm die and pressed into a circular pellet at ∼3 tons of pressure. The pellets were transferred to individual fused silica tubes with an inner diameter of 7 mm. These tubes were sealed under a vacuum of <100 mTorr to retain a rough length of 8 cm and then heated in a furnace (Thermoscientific Thermolyne 1400) at 550 °C for 20 h. After the reaction, the crystals were hammered and ball milled for 1 h to obtain fine FeGe powder.

CVD growth of Fe_3GeTe_2 (FGT) nanoplates

To synthesize FGT nanoplates, 1 μL of isopropyl alcohol (IPA) was used to spread 5 mg FeGe powder to a criss cross pattern on a Si/SiO_2 wafer (see Figure S2a). 50 mg Te powder (Alfa Aesar), 50 mg FeI_2 powder (Alfa Aesar), and the Si/SiO_2 substrate was put at the upstream end, center, and the downstream end of a 6-cm long alumina boat, respectively. The boat was kept at the center of a tube furnace in a home-built CVD setup, which was preheated to 450 °C under 250 sccm Ar and 5 sccm H_2 flow at 815 Torr. After a 10 min reaction, the furnace was opened and cooled down naturally. The resultant Fe_3GeTe_2 nanoplates were found on the substrate with well-shaped hexagons and triangles and lateral sizes reaching 15 micrometers and thickness down to 20 nm.

Characterizations of Fe_3GeTe_2 nanoplates

Optical microscope (Olympus BX51M), atomic force microscope (Agilent 5500), and Confocal Raman spectroscope (LabRAM Aramis Confocal Raman) were used to characterize the as-grown Fe_3GeTe_2 nanoplates on Si/SiO_2 substrates. Scanning electron microscopy (SEM) images were collected on a Zeiss SUPRA 55VP field emission scanning electron microscope at a typical accelerating voltage of 2–5 kV for imaging. Electron dispersive spectroscopy (EDS) spectra were collected on the same microscope by using an accelerating voltage of 22 kV and a Thermo Scientific UltraDry EDS detector.

Reflective magnetic circular dichroism (RMCD) measurements

The substrate with grown products was stored inside an Ar glovebox (H_2O< 0.01 ppm, O_2< 0.01 ppm) before the RMCD measurements and quickly transferred to the cryostat. The RMCD measurements were performed in an optical cryostat (Quantum Design, OptiCool) with a base temperature of 1.7 K and out-of-plane magnetic fields up to 7 T. A 632.8 nm He–Ne probe laser was intensity-modulated at 773 Hz by a mechanical chopper. The polarization was modulated using a photoelastic modulator (PEM; Hinds Instruments) operating at f = 50 kHz with a

retardation amplitude of π, followed by a quarter-wave plate to alternate between left- and right-circularly polarized light. The probe power was kept below 3 μW and focused to a ~2 μm spot with a 20× objective. The reflected signal was analyzed by a two-channel lock-in amplifier (OD1022D): the differential reflectance ΔR was extracted at the second harmonic of the PEM (2f), and the average reflectance R at the chopper frequency. The RMCD signal is defined as ΔR/R. Atomic force microscopy (AFM) imaging was performed on the same Fe_3GeTe_2 nanoplates examined by RMCD after the RMCD measurements in a flowing nitrogen atmosphere with humidity <5% to minimize the air exposure of the samples.

TEM

Electron transparent STEM samples of each Fe_3GeTe_2 sample were prepared on a Thermo Fisher Scientific Helios G4 UX focused ion beam using the standard lift-out method. Samples were thinned to <30 nm with 2-kV Ga ions, followed by a final polish at 1 kV to reduce the effects of surface damage. All specimens were stored in vacuum to prevent possible degradation in air.

The Scanning Transmission Electron Microscopy (STEM) experiment was performed using Thermo-Fisher FEI Spectra 300 TEM with ultra-high-brightness cold field emission gun (C-FEG) and spherical aberration correction (aka. "Andromeda" at Cornell University). The STEM resolution is 58 pm with probe current = 75 pA. The High-annular angle dark field (HAADF) image was collected with C2 aperture = 70 um, camera length = 94 mm and convergence angle = 30 mrad with 60 mrad inner and 200 mrad out collection angles. The HAADF image stacks were taken with 2048×2048 pixels with 200 ns dwell times to 30 frames, then the image stacks were sum to one frame after rigid-registration to enhance signal-to-noise ratio (SNR) and reduce the effect of sample drift.

The electron ptychography data was collected using Electron Microscopy Pixelated Array Detector Generation 2 (EMPAD-G2), with 256×256 pixels in real space with 100 μs dwell time. The 3D information was reconstructed from multislice algorithms, with one single x-y scan, that is recorded within a few seconds.

Quantification of Intercalated Fe Content in the vdW gaps

The phase of a reconstructed electron ptychography image is approximately proportional to the electrostatic potential projected through the specimen thickness. Because the electrostatic potentials contributed by individual atoms are additive, the phase signal integrated around an Fe column is approximately proportional to the number of Fe atoms contained in that column. We therefore used the integrated phase signal, rather than the maximum phase at the center of the column, as a measure of the relative Fe content.

To quantify the Fe content, we first selected the crystalline interior of the reconstructed volume by summing the bulk ten 1-nm-thick MEP slices, excluding the surface-damaged or oxidized regions. This summed image is shown in Figure S7. The blue dots overlaid on the Figure S7 left image

mark the Fe atomic columns used for quantitative intensity extraction and the green arrows indicate representative rows of Fe columns to guide the eye. The corresponding Fe-column intensity map in Figure S7 (right) shows the extracted signal at each Fe-column position, where darker markers indicate higher Fe-column signal and lighter markers indicate lower Fe-column signal.

The unit-cell Fe positions were refined by two-dimensional Gaussian fitting. Because the interstitial Fe columns were weaker, their positions were refined using a matched filter followed by a weighted-centroid calculation. These procedures were used only to determine the column positions. All Fe signals were measured from the original, unfiltered ptychographic phase image using the same procedure.

For each Fe column, the local background was defined as the median phase within an annulus extending from the column center. The background-subtracted column signal was then calculated by summing all pixels within a radius of 4 pixels:

Column signal = sum of (pixel phase − local background) within the 4-pixel-radius disk.

The extracted Fe-column signals are summarized in histograms in Figure S8. The unit cell Fe columns are used as an internal reference and normalized to a median value of 1.0, with the assumption that these unit cell Fe sites are close to full occupancy, which is consistent with their strong and continuous intensity in the MEP depth profiles and with the FGT structure. With this reference, the intercalated Fe columns have an average normalized occupancy of approximately 0.4 and show a broader distribution, indicating a low and spatially inhomogeneous intercalated Fe population. The relative interstitial Fe content was calculated by dividing the total signal from all interstitial Fe columns by the total signal from all unit-cell Fe columns:

Interstitial-to-unit-cell Fe ratio

= total interstitial Fe column signal / total unit-cell Fe column signal

$= 8.896 \times 10^4 / 4.344 \times 10^5$

= 0.2048.

Because the crystallographic reference contains two Fe atoms, the corresponding interstitial Fe content is:

Interstitial Fe content = 2 × 0.2048 = 0.4096.

The measured phase signal corresponds to approximately 0.4 intercalated Fe atoms per Fe_3GeTe_2 formula unit. In other words, the chemical formula of the synthesized product is $Fe_{3.4}GeTe_2$.

This conversion assumes that the reconstructed phase is approximately linear with projected electrostatic potential and that each Fe atom contributes approximately the same integrated phase signal in unit cell and interstitial Fe columns.

Additional Figures and Tables

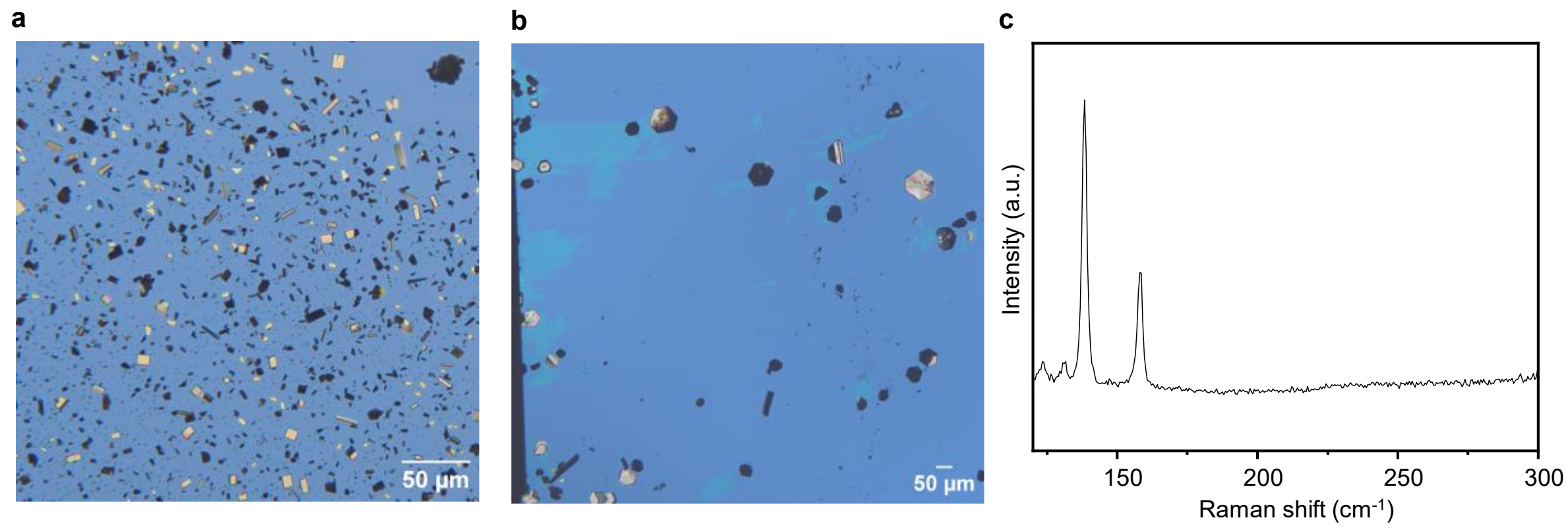

Figure S1. Optical microscope images of the byproducts near the edge of the wafer. **a**) Rectangular nanoplates of tetragonal FeTe phase and long nanowhiskers of orthorhombic $FeTe_2$. **b**) Hexagonal $FeTe_2$ nanoplates. **c**) Raman spectrum of the thick hexagonal $FeTe_2$ nanoplates shown in (**b**), which matches the reported Raman peaks of hexagonal $FeTe_2$.[2]

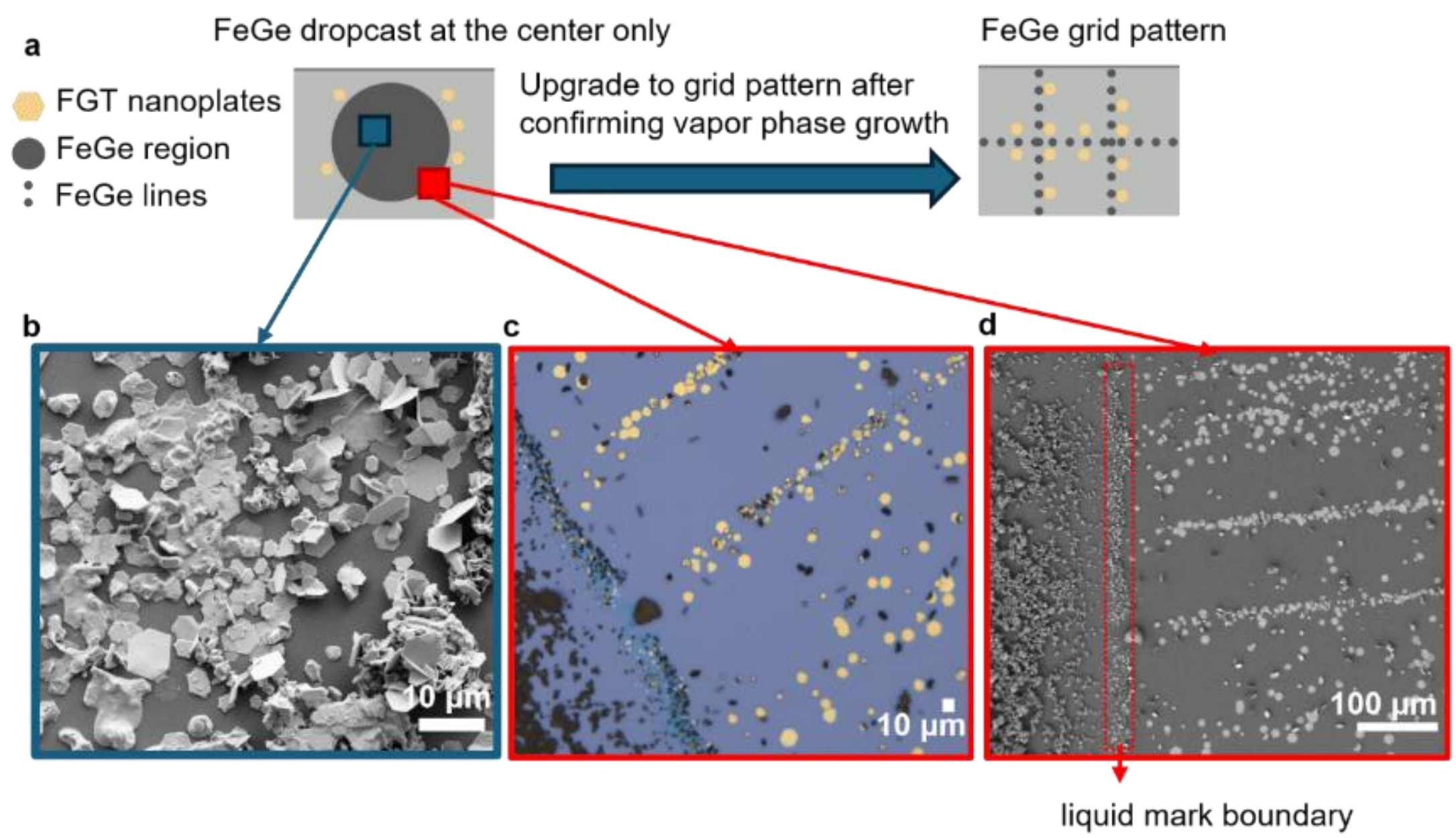

Figure S2. Illustration of spatial distribution of reaction products**. a**) Illustration of the growth substrate with FeGe precursor dropcast at center only in comparison with FeGe grid pattern. **b**) SEM image of Fe_3GeTe_2 nanoplate clusters inside the FeGe seeded region in blue box region of (**a**). **c,d**) Optical and SEM image of the seeded boundary region in red box region of (**a**). Small Fe_3GeTe_2 plates inside the boundary and large hexagonal Fe_3GeTe_2 plates outside the boundary, confirming the vapor phase growth mechanism.

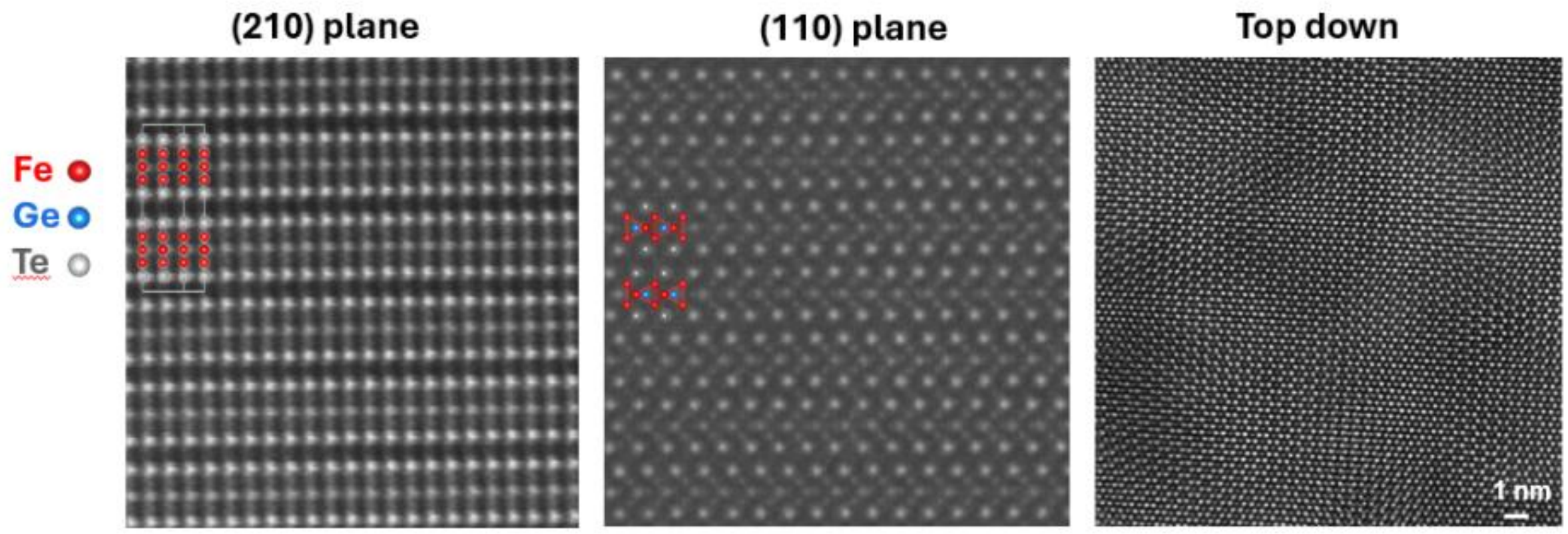

Figure S3. Top-down STEM images and cross-sectional STEM images of (210) plane and (110) plane of Fe_3GeTe_2 nanoplates with schematic models of corresponding planes overlayed.

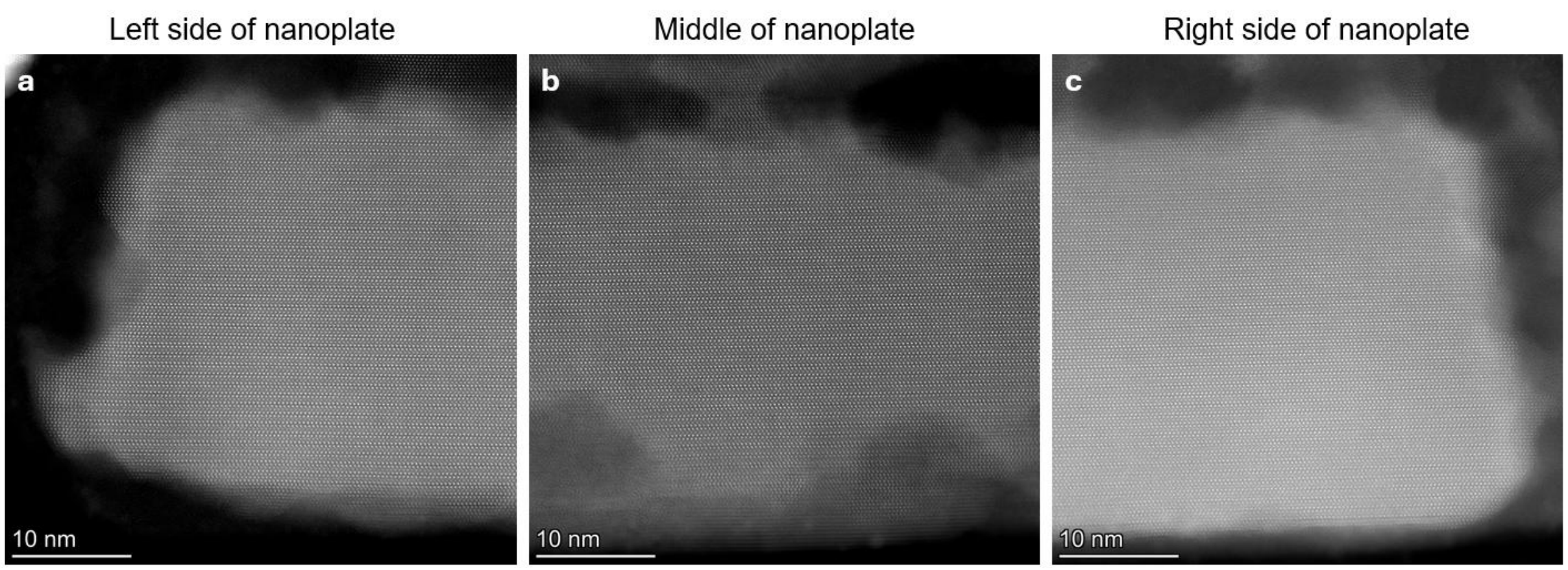

Figure S4. Cross-sectional STEM images of Fe_3GeTe_2 nanoplates with larger field-of-view at different regions.

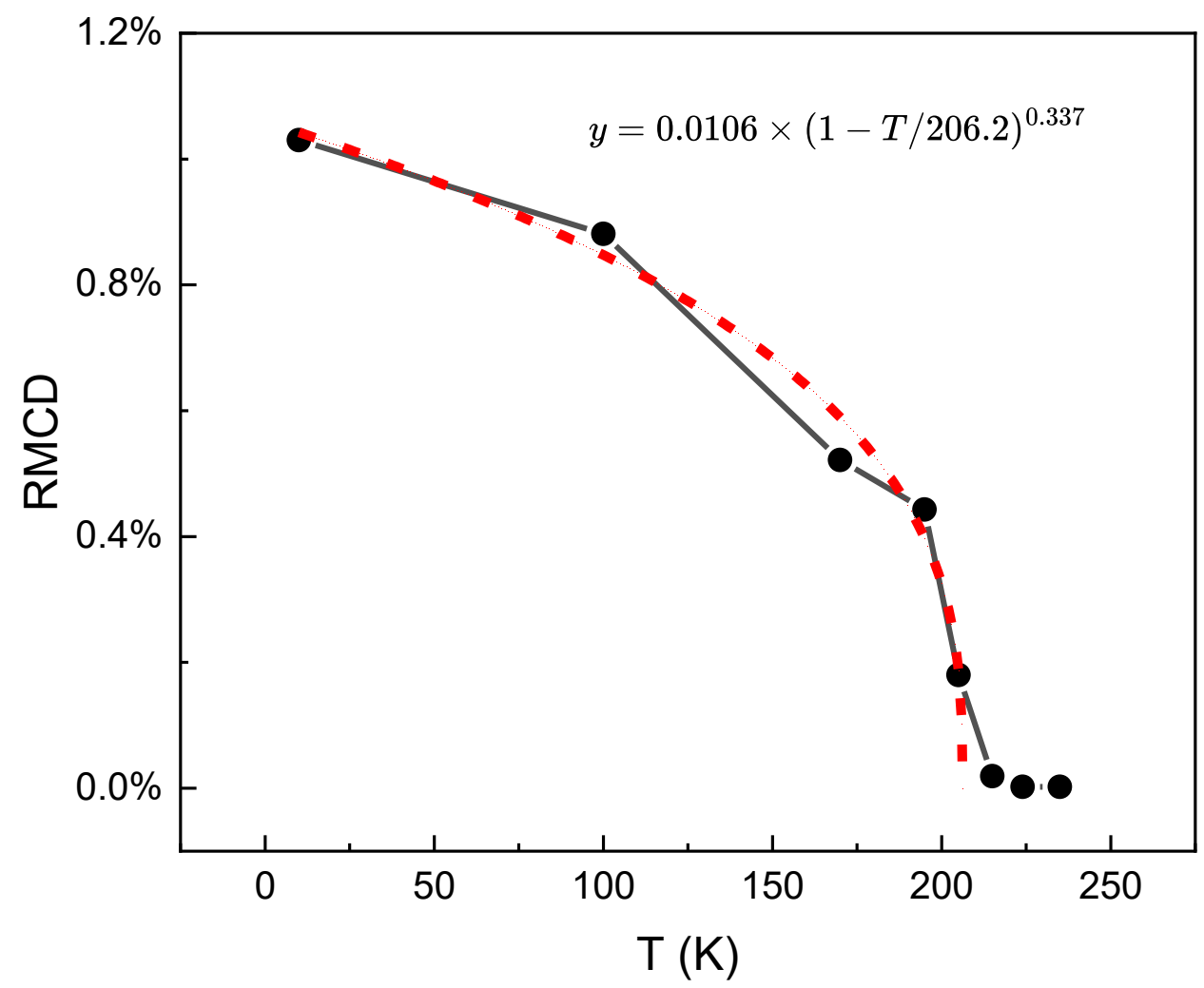

Figure S5. Temperature dependence of the RMCD signal of Fe_3GeTe_2 Flake 1 with a model fit used to extract the Curie temperature for this Fe_3GeTe_2 nanoplates.

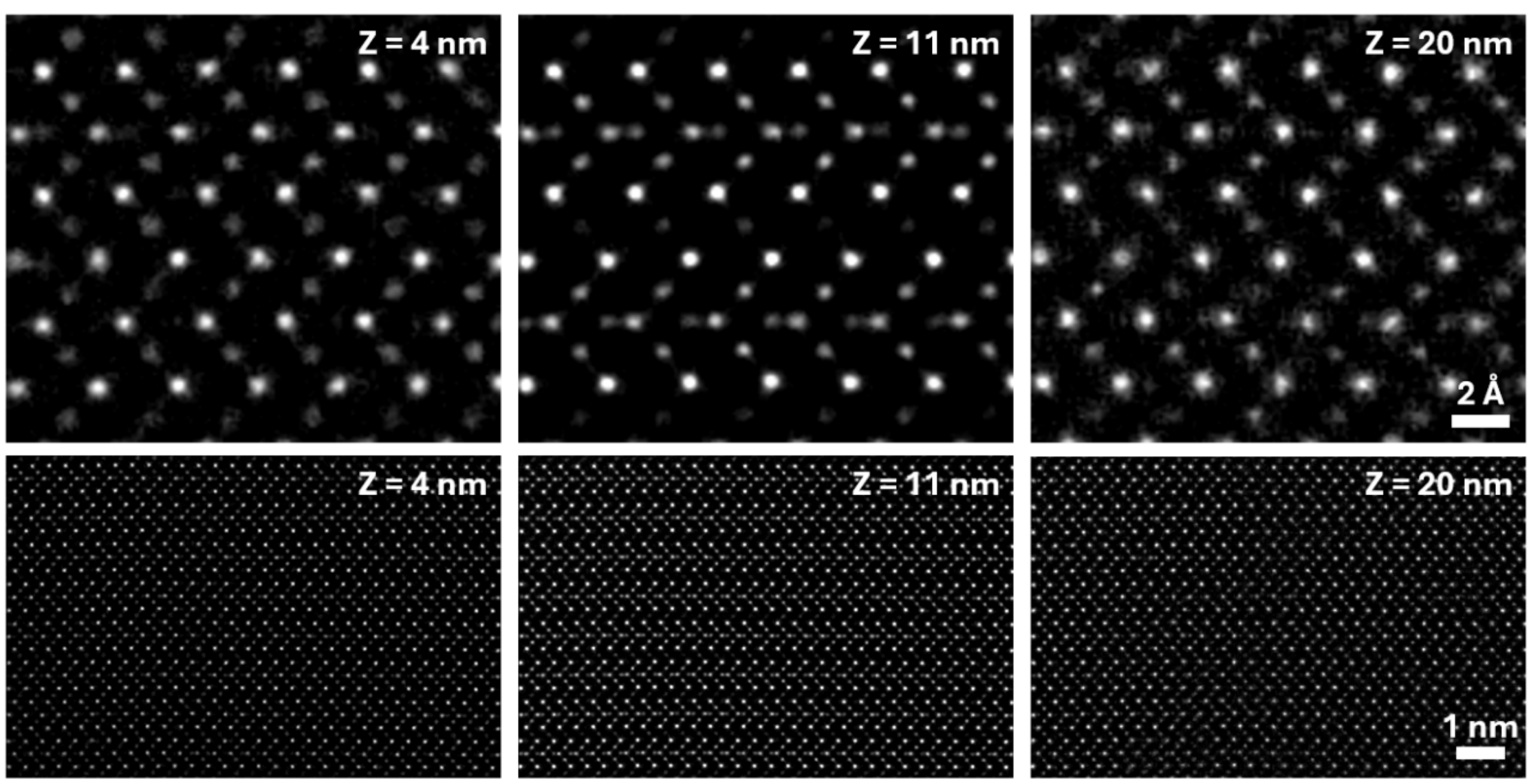

Figure S6. Cross sectional MEP images of Fe_3GeTe_2 nanoplates along [110] axis. These slices at different steps are from a single MEP dataset, a single x-y scan within a few seconds.

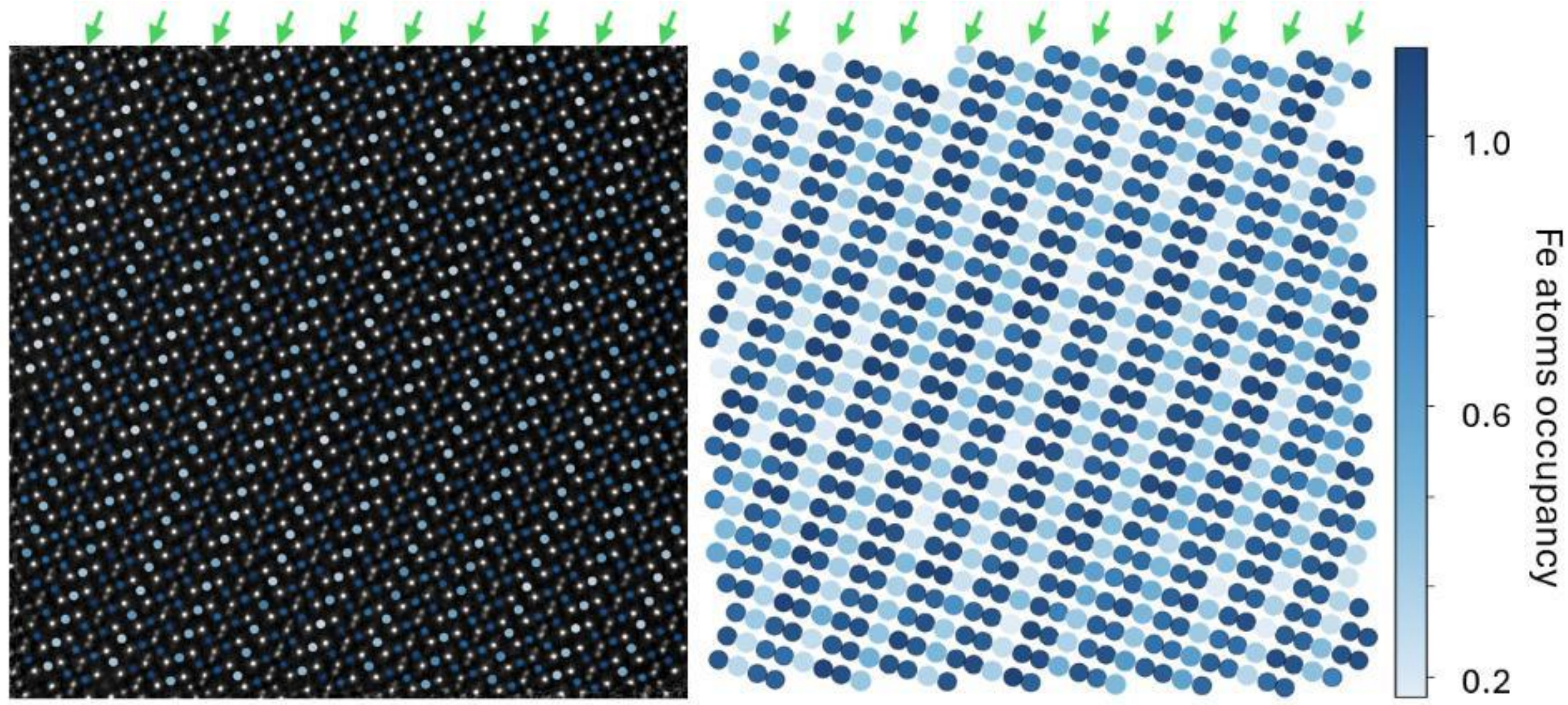

Figure S7. Intensity map derived from multislice electron ptychography.

The figure shows a two-dimensional map of the reconstructed signal obtained by summing middle 10 1-nm slices from a multislice electron ptychography reconstruction, which reconstructs the projected electrostatic potential of the material (dominated by the nuclear potential). Here, the 10 x 1-nm slices were added together to give an integrated, projected view of the sample through its thickness.

Left: summed reconstructed image. Bright features correspond to strongly scattering atomic columns or local regions with larger reconstructed phase signal. The blue dots mark the Fe atomic columns selected for intensity extraction. The green arrows indicate representative Fe-column rows to guide the eye and show which columns were quantified.

Right: simplified Fe-column intensity map of the same field of view, without overlaid on the image. Each dot represents one Fe atomic-column position. The color of each dot gives the summed reconstructed intensity at that position. Light blue/white markers indicate lower summed intensity, while darker blue markers indicate higher summed intensity, as shown by the color bar. The values were converted to relative Fe occupancy by setting the average signal of the each unit cell Fe sites in the FGT lattice to 1.0, corresponding to full occupancy. Therefore, lower values indicate Fe columns with fewer Fe atoms relative to the fully occupied unit cell Fe sites.

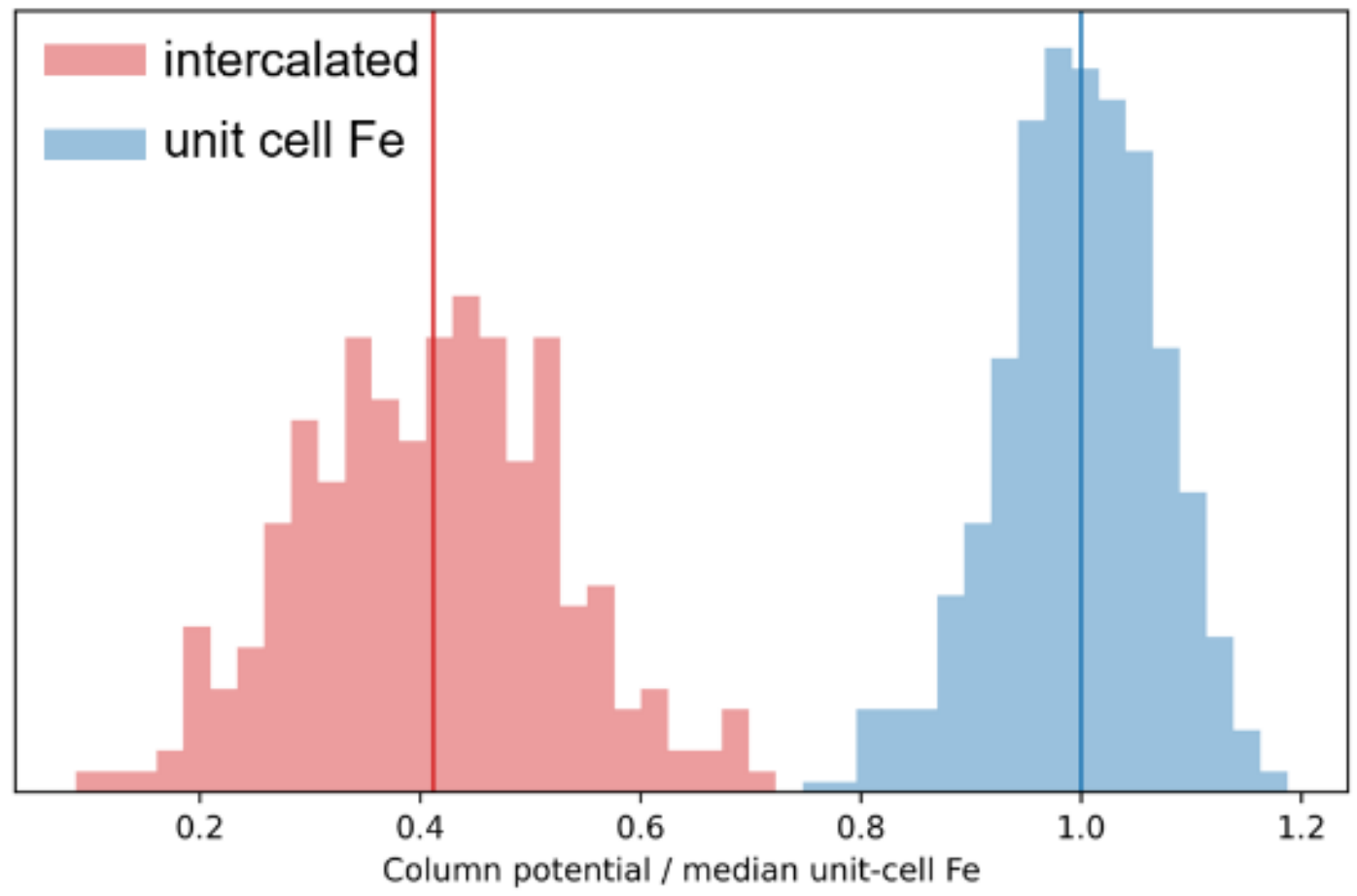

Figure S8. Quantification of intercalated Fe occupancy from the Fe-column intensity map in Figure S7.

Histogram of the normalized Fe-column signal extracted from Figure S7. The blue histogram shows the unit cell Fe columns in the FGT lattice, and the red histogram shows the intercalated Fe columns in the van der Waals gaps. The column signal was normalized by the median signal of the unit cell Fe columns, which are taken as fully occupied Fe sites with an occupancy of 1.0. With this normalization, the intercalated Fe columns have an average relative occupancy of approximately 0.4, corresponding to a chemical formula of $Fe_{3.4}GeTe_2$. The larger spread of the intercalated Fe histogram indicates that the intercalated Fe atoms are distributed inhomogeneously, with larger column-to-column variation than that observed for the unit cell Fe columns.

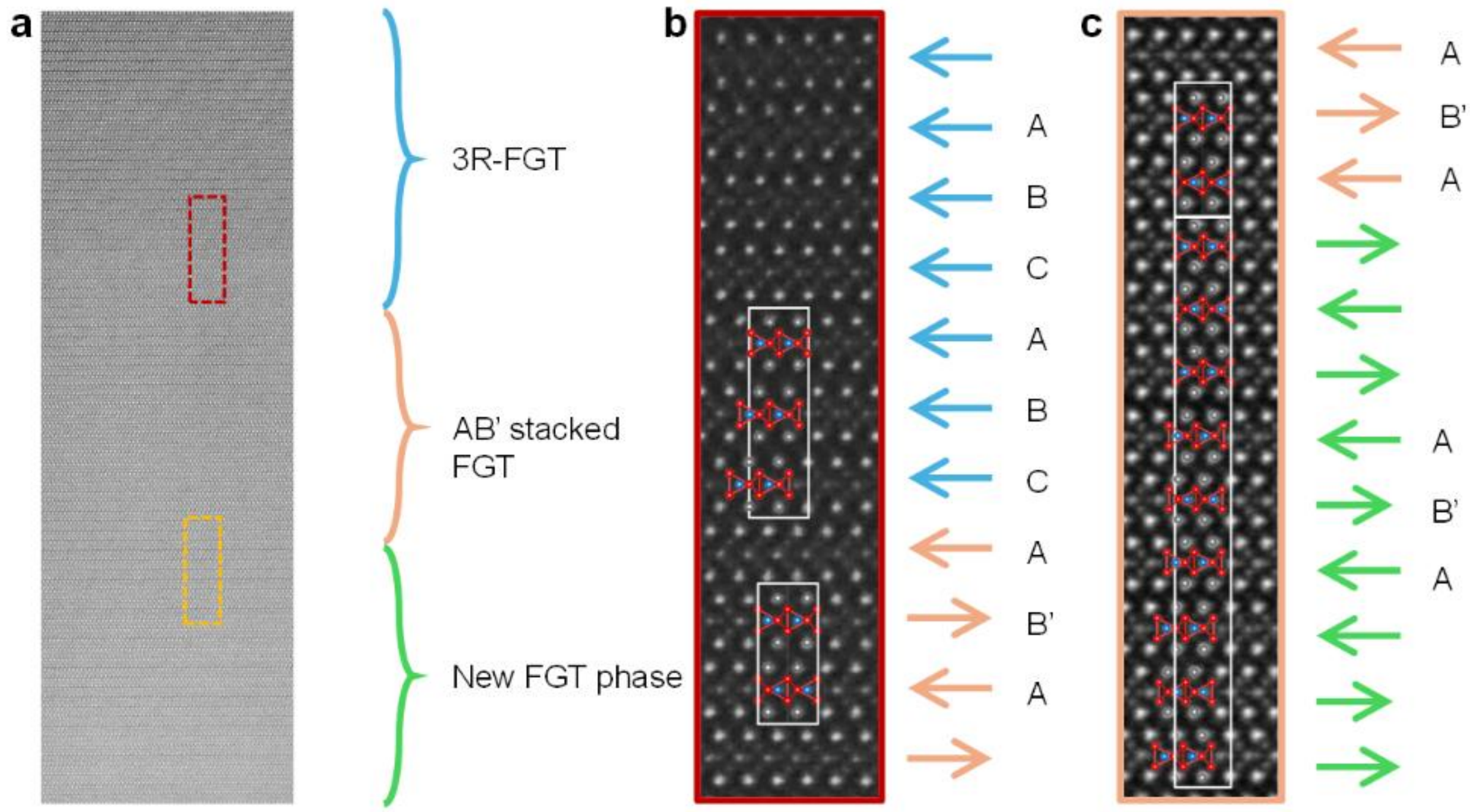

Figure S9. Structural layer stacking anomalies in Fe_3GeTe_2 (FGT) nanoplates. **a**) Representative cross-sectional HAADF-STEM image of an FGT nanoplate exhibiting distinct stacking phases along the out-of-plane direction. **b**) Magnified view of the region indicated by the dashed blue box in **a**, highlighting the interface between the 3R and AB' stacking sequences. **c**) High-resolution image of the dashed orange box in **a**, showcasing the transition from AB' stacking to a complex, 9-layer macroscopic 3R-like phase. White boxes illustrate the unit cells.

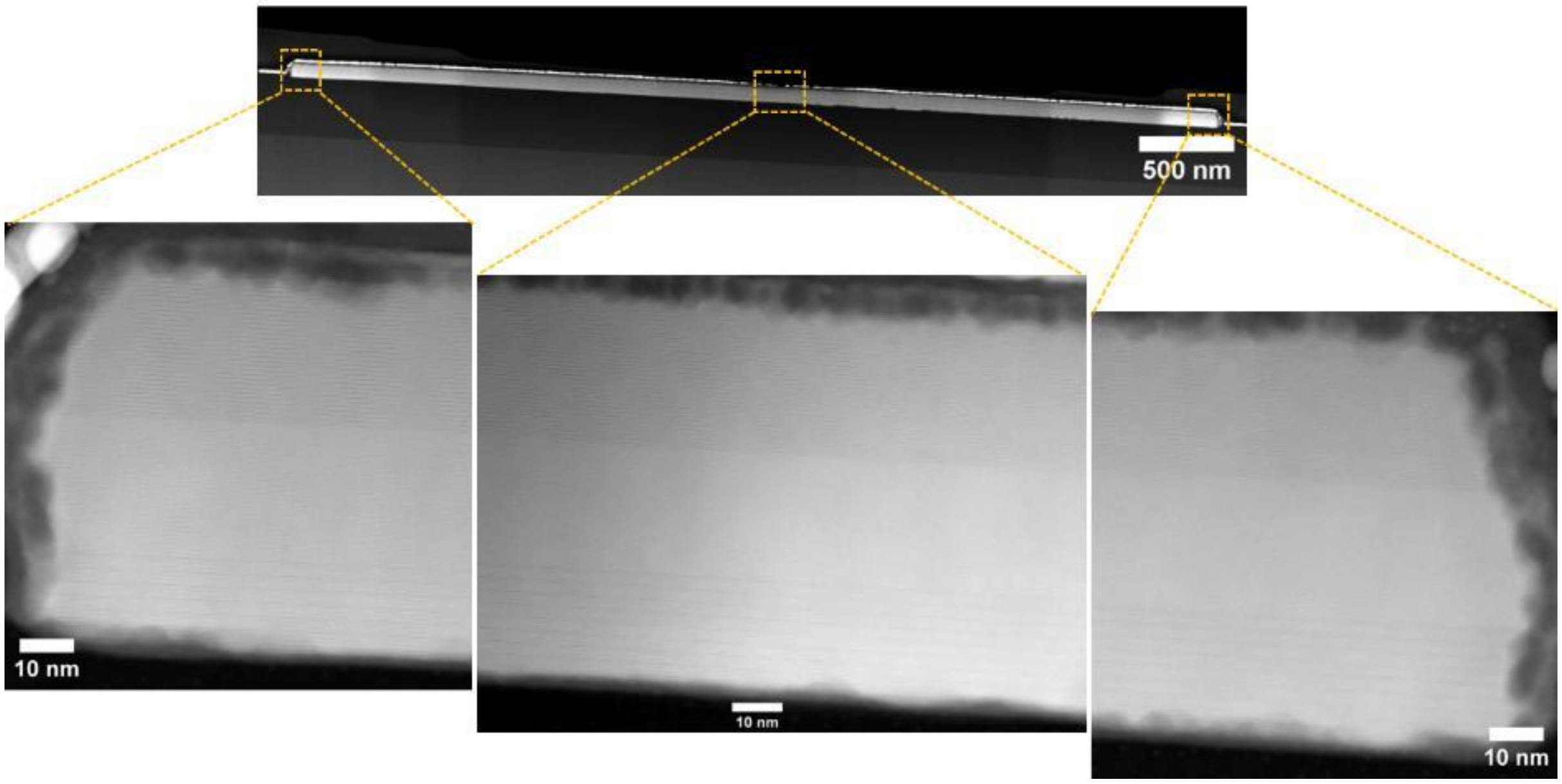

Figure S10. Cross-sectional STEM images of Fe_3GeTe_2 (FGT) nanoplates along [110] axis at different regions, showing lateral homogeneity and three different (stacking) FGT phases with vertical boundaries.

Table S1. Magnetic properties of Fe_3GeTe_2 in comparison with the values reported in the literature.

Samples	Coercive field	Curie temperature	Technique	Reference
FGT nanoplates (38 nm)	~4500 Oe at 100 K	~206 K	RMCD	This work
Exfoliated FGT film (48 nm)	~3000 Oe at 105 K	~210 K	RMCD	[3]
Exfoliated FGT film (50L/ 40 nm)	~1700 Oe at 100 K	~180 K	Hall measurement	[4]
MBE-grown FGT thin film (20 nm)	~2000 Oe at 160 K	~220 K	VSM	[5]
MBE-grown FGT thin film (8 nm)	~3100 Oe At 100 K	~216 K	Hall measurement	[6]
MBE-grown $Fe_{3.18}GeTe_2$ (10 nm)	~2500 Oe at 100 K	~215 K	Hall measurement	[7]
Exfoliated FGT flake (40 nm)	~3800 Oe at 100 K	~200 K	Hall measurement	[8]
Exfoliated FGT flake (~ 7 nm)	~1700 Oe at 80 K	~165 K	RMCD	[9]
CVD grown FGT flake (13 nm)	~2800 Oe at 100 K	~200 K	RMCD	[10]
Exfoliated FGT flakes (50 nm)	~500 Oe at 110 K	~180 K	Hall measurement	[11]
FGT bulk single crystal	~45 Oe at 2 K	~183 K	SQUID	[12]
MBE grown FGT film (20 nm)	~2500 Oe at 100 K	~250 K (not from fitting)	MCD	[13]
Exfoliated FGT flake (49 nm)	~1000 Oe at 100 K	~194 K	Hall measurement	[14]

References cited in the Supporting Information:

1. Stolt, M. J.; Sigelko, X.; Mathur, N.; Jin, S. Chemical Pressure Stabilization of the Cubic B20 Structure in Skyrmion Hosting $Fe_{1-X}Co_xGe$ Alloys. *Chemistry of Materials* **2018**, *30*, 1146–1154. https://doi.org/10.1021/acs.chemmater.7b05261.
2. Chen, S., Liu, H., Chen, F., Zhou, K., & Xue, Y. Synthesis, Transfer, and Properties of Layered $FeTe_2$ Nanocrystals. *ACS Nano* **2020**, *14*, 11473–11481. https://doi.org/10.1021/acsnano.0c03863
3. Fei, Z., Huang, B., Malinowski, P., Wang, W., Song, T., Sanchez, J., Yao, W., Xiao, D., Zhu, X., May, A. F., Wu, W., Cobden, D. H., Chu, J. H., & Xu, X. Two-dimensional itinerant ferromagnetism in atomically thin Fe_3GeTe_2. *Nat. Mater.* **2018,** *17*, 778–782. https://doi.org/10.1038/s41563-018-0149-7
4. Deng, Y., Yu, Y., Song, Y., Zhang, J., Wang, N. Z., Sun, Z., Yi, Y., Wu, Y. Z., Wu, S., Zhu, J., Wang, J., Chen, X. H., & Zhang, Y. Gate-tunable room-temperature ferromagnetism in two-dimensional Fe_3GeTe_2. *Nature* **2018**, *563*, 94–99. https://doi.org/10.1038/s41586-018-0626-9
5. Xie, W.; Zhang, J.; Bai, Y.; Liu, Y.; Wang, H.; Yu, P.; Li, J.; Chang, H.; Wang, Z.; Gao, F.; et al. Air Stability and Composition Evolution in van Der Waals Fe_3GeTe_2. *APL Mater.* **2024**, *12*, 031102. https://doi.org/10.1063/5.0194520.
6. Liu, S., Yuan, X., Zou, Y. et al. Wafer-scale two-dimensional ferromagnetic Fe_3GeTe_2 thin films grown by molecular beam epitaxy. *npj 2D Mater Appl* **2017**, *1*, 30. https://doi.org/10.1038/s41699-017-0033-3
7. Liu, S.; Li, Z.; Yang, K.; Zhang, E.; Narayan, A.; Zhang, X. et al. Tuning 2D Magnetism in $Fe_{3+X}GeTe_2$ Films by Element Doping. *Natl. Sci. Rev.* **2022**, *9*, nwab117. https://doi.org/10.1093/nsr/nwab117
8. Wu, Y.; Yang Wang, B.; Yu, Y.; Li, Y.; Ribeiro, H. B.; Wang, J.; Xu, R.; Liu, Y.; Ye, Y.; Zhou, J.; et al. Interlayer Engineering of Fe3GeTe2: From 3D Superlattice to 2D Monolayer. *Proc. Natl. Acad. Sci. U. S. A.* **2024**, *121*, e2314454121. https://doi.org/10.1073/pnas.2314454121.
9. Tu, Z.; Xie, T.; Lee, Y.; Zhou, J.; Admasu, A. S.; Gong, Y.; Valanoor, N.; Cumings, J.; Cheong, S. W.; Takeuchi, I.; et al. Ambient Effect on the Curie Temperatures and Magnetic Domains in Metallic Two-Dimensional Magnets. *NPJ 2D Mater. Appl.* **2021**, *5*, 62. https://doi.org/10.1038/s41699-021-00242-z.

10. Nair, G. K. R.; Zhang, Z.; Hou, F.; Abdelaziem, A.; Xu, X.; Yang, S. W. Q.; Zhang, N.; Li, W.; Zhu, C.; Wu, Y.; et al. Phase-Pure Two-Dimensional FexGeTe2 Magnets with near-Room-Temperature T C. *Nano Res.* **2022**, *15*, 457–464. https://doi.org/10.1007/s12274-021-3502-0.
11. Wang, Y.; Wang, C.; Liang, S. J.; Ma, Z.; Xu, K.; Liu, X.; Zhang, L.; Admasu, A. S.; Cheong, S. W.; Wang, L.; et al. Strain-Sensitive Magnetization Reversal of a van Der Waals Magnet. *Advanced Materials* **2020**, *32*, 2004533. https://doi.org/10.1002/adma.202004533.
12. Bera, S.; Pradhan, S. K.; Pal, R.; Pal, B.; Bera, A.; Kalimuddin, S.; Das, M.; Roy, D. S.; Afzal, H.; Pal, A. N.; et al. Enhanced Coercivity and Emergent Spin-Cluster-Glass State in 2D Ferromagnetic Material, Fe3GeTe2. *J. Magn. Magn. Mater.* **2023**, *583*, 171052. https://doi.org/10.1016/j.jmmm.2023.171052.
13. Zhou, W.; Bishop, A. J.; Zhu, M.; Lyalin, I.; Walko, R.; Gupta, J. A.; Hwang, J.; Kawakami, R. K. Kinetically Controlled Epitaxial Growth of Fe_3GeTe_2 van Der Waals Ferromagnetic Films. *ACS Appl. Electron. Mater.* **2022**, *4*, 3190–3197. https://doi.org/10.1021/acsaelm.2c00185.
14. Tan, C.; Lee, J.; Jung, S. G.; Park, T.; Albarakati, S.; Partridge, J.; Field, M. R.; McCulloch, D. G.; Wang, L.; Lee, C. Hard Magnetic Properties in Nanoflake van Der Waals Fe3GeTe2. *Nat. Commun.* **2018**, *9*, 1554. https://doi.org/10.1038/s41467-018-04018-w.
